# Wuzhishan miniature pig-derived intestinal 2D monolayer organoids to investigate the enteric coronavirus infection

**DOI:** 10.3389/fvets.2024.1457719

**Published:** 2024-09-25

**Authors:** Yuanyuan Liu, Ning Yang, Chen Tan, Yunhang Zhang, Shuai Gao, Yifei Cai, Yue Zhang, Yuguang Fu, Guangliang Liu, Yang Li

**Affiliations:** ^1^Key Laboratory of Tropical Animal Breeding and Disease Research, Institute of Animal Husbandry and Veterinary Medicine, Hainan Academy of Agricultural Sciences, Haikou, China; ^2^State Key Laboratory for Animal Disease Control and Prevention, Lanzhou Veterinary Research Institute, Chinese Academy of Agricultural Sciences, Lanzhou, China; ^3^College of Veterinary Medicine, Xinjiang Agricultural University, Ürümqi, China; ^4^Molecular and Cellular Epigenetics (GIGA), University of Liège, Liège, Belgium; ^5^Human Nutrition and Health Group, VLAG, Wageningen University & Research, Wageningen, Netherlands

**Keywords:** intestinal organoids, organoids monolayers, RNA-seq, TGEV, Wuzhishan pigs

## Abstract

Intestinal organoids are valuable tools for investigating intestinal physiology and pathology *ex vivo*. In previous studies, intestinal organoids of commercial pigs have been developed. Here, we established intestinal organoids derived from Wuzhishan miniature pigs (WZS pigs), a unique kind of pig in the Hainan province of China. Three-dimensional (3D) intestinal organoids and organoid monolayers were developed and assessed. Furthermore, the susceptibility of organoid monolayers of WZS pigs to transmissible gastroenteritis virus (TGEV) was demonstrated. An RNA-seq analysis revealed that the TGEV infection stimulated antiviral and inflammatory immune responses in organoid monolayer models. The study implied the transmission risk of swine enteric coronavirus on WZS pigs and provided useful tools for further research on WZS pigs as laboratory miniature pig models.

## Introduction

Miniature pigs are potential laboratory animals to study human diseases because they can mimic humans physiologically and metabolically to a large extent. Wuzhishan miniature pigs (WZS pigs) are one of the most important Chinese domestic pig breeds, which belong to the 138 China national genetic resources protected breeds list. WZS pigs have the advantages of resistance to coarse feeding and high temperature and humidity climate in tropical areas in Hainan province. Hence, WZS pigs are physically different from intensive commercial pig breeds. Moreover, due to their small size (with 30–35 kg adult weights), Wuzhishan miniature pigs are used in human health and veterinary research. Currently, WZS pigs are utilized to study diabetes (1), atherosclerosis (2), allergy (3), and xenograft (4).

With a continuous explosion of complex luminal microbiome and basolateral immune cells, intestinal epithelium plays a vital role in innate and adaptive immune responses. A physical *in vitro* model of intestinal epithelium can help to understand intestinal mucosal barrier development, nutrient uptake characteristics, and enteric pathogen infection in local WZS pigs. Furthermore, the WZS pig-derived *in vitro* model may give different insights into microbes–host interaction compared to the model derived from intensive commercial pig breeds.

Intestinal organoids are multicellular and self-organized models that can recapitulate multiple aspects of real intestinal epithelium (5). Intestinal organoids of humans and farm animals were established to investigate the function of the epithelial barrier, enteric pathogen invasion, and mucosal immune responses (6–8). In the previous study, we developed porcine intestinal organoids derived from intensive commercial pig breeds and investigated viral infection, antiviral response, and intestinal epithelial regeneration upon organoids (9, 10). In this study, to make better use of WZS pigs for interrogating pathogens–host interaction upon intestinal mucosa, we developed WZS pig-derived intestinal organoids. Furthermore, based on the 3D intestinal organoids, the 2D organoid monolayers were generated and utilized for assessing the enteric coronavirus infection. We found that 2D monolayers with less differentiation are susceptible to TGEV infection. Transcriptome analysis indicated that TGEV infection stimulates antiviral and inflammatory immune responses. Collectively, our studies established the intestinal organoids model of the WZS pigs and reported its susceptibility to TGEV, which suggested the potential risk of swine enteric coronavirus infection in WZS pigs.

## Materials and methods

### Establishment and passage of intestinal 3D intestinal organoids

Intestinal tissues of the WZS pigs used in this study were obtained from adult WZS pigs of the National WZS Pig Farm in Hainan Province. Tissues of adult Yorkshire pigs were obtained from the slaughterhouse of Luoniushan Group Co., Ltd. Crypts were isolated from the ileum as described previously (9). Crypts were resuspended in 50% Matrigel (Corning, United States) mixed with DMEM/F12 medium and seeded into a 24-well plate (50 μL for each dome). After solidification of the dome (in an incubator under 37°C for 15 min), IntestiCult™ Organoid Growth Medium (OGM, STEMCELL Technologies, Canada) with 10 μM Y-27632 (STEMCELL Technologies, Canada) and 1X penicillin–streptomycin–amphotericin B solution (Solarbio, China) were added into 24-well plate (750 μL/well). Half-medium change was performed every 2 days. After 3–7 days of culture in a CO_2_ incubator (at 37°C), 3D intestinal organoids were formed. 3D organoids were passaged as described previously (9).

### Generation of 2D intestinal organoids and evaluation of epithelial integrity

Transwell inserts (with 0.4 μm pore, 6.5 mm, Corning, United States) were pre-coated with DMEM/F12 medium (Sigma, Germany) containing 2% Matrigel at 37°C for 2 h. Then, the DMEM/F12-Matrigel solution was removed. Well-established 3D intestinal organoids were collected from Matrigel domes and dissociated by TrypLE™ Express (Gibico, United States) at 37°C for 10 min. After being centrifuged for 5 min (200 × g, 4°C), single cells were resuspended with OGM or IntestiCult™ Organoid Differentiation Medium (ODM, STEMCELL Technologies, Canada) and seeded into pre-coated Transwell inserts (1.5 × 10^5^ cells/well), 100 μL single cell suspension on top and 500 μL medium on bottom or 24-well cell culture plates (Corning, United States). Transepithelial electrical resistance (TEER) was detected via Millicell ERS-2 (Millipore, United States). Confluence of epithelial cells can be observed in 48 to 72 h post-seeding.

### TGEV infection

The TGEV Miller strain was stored in our lab and propagated on swine testicular (ST) cells. For infecting organoid monolayers with TGEV, confluent organoid monolayers (supported by OGM) in 24-well plates and Transwell inserts were washed three times with PBS buffer. Organoid monolayers were incubated with TGEV (MOI = 5) for 2 h at 37°C with 5% CO_2_. The supernatant from ST cells lysate was used as Mock control. TGEV and ST cell lysate were diluted in DMEM/F12 medium. After TGEV infection, medium change was performed following a PBS wash. The TGEV in the supernatant was then titrated in ST cells using a TCID_50_ assay at the indicated time points.

### Immunostaining

Organoid monolayers (supported by OGM) in Transwell inserts were fixed with 4% paraformaldehyde for 15 min and permeated with 0.4% Triton X-100 (Solarbio, China) for 15 min at room temperature (RT). After 1 h of blocking with 5% BSA (Solarbio, China) at RT, monolayers were incubated overnight at 4°C with antibodies against Ki67 (ab15580, Abcam, United Kingdom), Chromogranin A (sc-393941, CGA, Santa Cruz, United States), lysozyme (ab2408, LYZ, Abcam, United Kingdom), ZO1 (ab221547, Abcam, United Kingdom), and TGEV N (lab-made) at the manufacturer-recommended dilutions. Organoid monolayers were incubated with secondary antibodies, goat anti-mouse IgG (ab150113, Abcam, United Kingdom) or goat anti-rabbit IgG (ab150077, Abcam, United Kingdom), at 37°C for 1 h at RT. Cell nuclei were stained with 2-(4-amidinophenyl)-6-indolecarbamidine dihydrochloride (DAPI, Beyotime Biotechnology, China) for 5 min at RT. Polyester membranes from Transwell inserts with organoid monolayers were cut out and mounted in a glass slide. Organoid monolayers were imaged using a ZEISS LSM 900 laser scanning confocal microscope (Carl Zeiss, Germany).

### RNA isolation and reverse transcription

RNA of 3D intestinal organoids or organoid monolayers of WZS pigs was extracted using the TransZol Up Plus RNA Kit (Transgene, China). For RT-qPCR detection, cDNA was prepared using the HiScript II 1st Strand cDNA Synthesis Kit (Vazyme, China). The RT-qPCR was conducted using ChamQ SYBR qPCR Master Mix (Vazyme, China). The parameters were 95°C for 30 s for initial denaturation; 40 cycles of 95°C for 10 s and 60°C for 30 s, which was followed by a dissociation curve segment (95°C, 15 s; 60°C, 60 s; 95°C, 15 s). Bio-Rad CFX96 was used for RT-qPCR detection. The primer sequences used for the RT-qPCR are listed in Supplementary Table S1.

### Bulk RNA-sequencing

Total RNA was extracted using a TRIzol reagent Kit (Invitrogen, United States), and the quality of the RNA was assessed using an Agilent 2,100 Bioanalyzer and RNase-free agarose gel electrophoresis. mRNA was enriched using Oligo(dT) beads and fragmented into short fragments using a fragmentation buffer. mRNA was transcribed into cDNA using the NEBNext Ultra RNA library Prep Kit for Illumina (NEB, United States). The Illumina NovaSeq 6,000 was used for RNA sequencing by Gene Denovo Biotechnology Co., Ltd., (Guangzhou, China). Reads obtained from the sequencing were filtered using fastp (version 0.18.0). The rRNA-mapped reads were removed according to the short reads alignment tool Bowtie2. A total of 42–69 million reads were sequenced, and 83 to 86% of reads were mapped to the genome of WZS pigs (GCA_000325925.2) using HISAT2 2.1.0. The mapped reads of each sample were assembled using StringTie v1.3.1. TPM value was calculated to quantify its expression abundance and variations using RSEM software. Differential expression analysis was performed using DESeq2 software between the two groups. The genes with the FDR below 0.05 and absolute fold change ≥ 2 were considered differentially expressed genes (DEGs). Raw sequencing data can be accessed on the BioProject database (accession: PRJNA1062093). Bioinformatic analysis was performed using Omicsmart, a dynamic real-time interactive online platform for data analysis.^1^

### Statistical analysis

All results are representative of three independent experiments. The data are presented as means ± standard error of the mean (SEM) and were analyzed using the two-tailed Student’s *t*-test or a one-way analysis of variance using GraphPad Prism 9.4.1 (GraphPad Software, United States). *p*-values of <0.05 were considered statistically significant and indicated as *p < 0.05, **p < 0.01, and ***p < 0.001.

## Results

### Establishment of 3D and 2D intestinal organoids derived from WZS pigs

**Figure 1:**
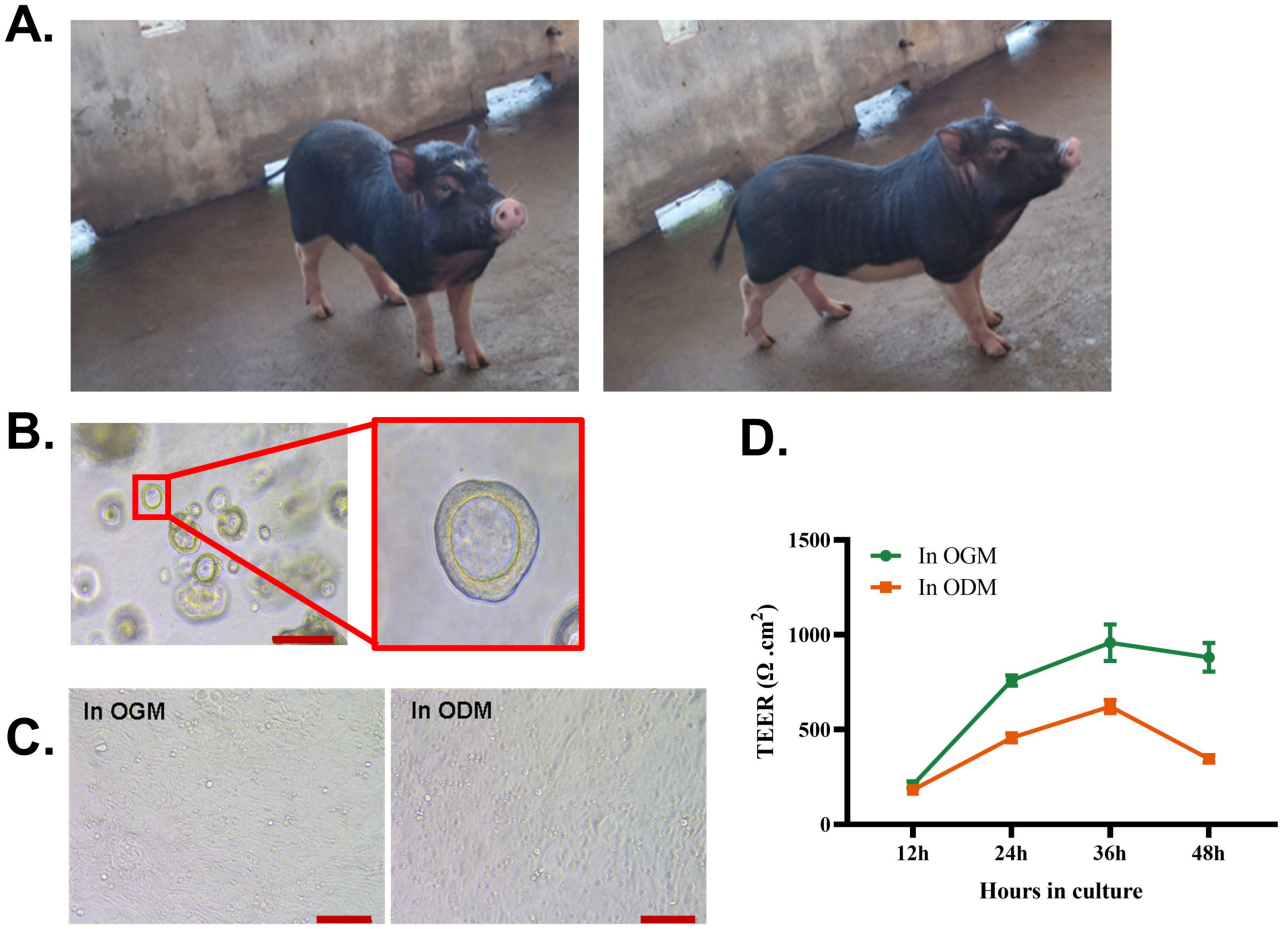
Establishment of 3D and 2D intestinal organoids derived from WZS pigs Representative photograph of adult WZS miniature pigs **(A)**. 3D intestinal organoids were established by isolating crypt cells from the ileum of adult WZS miniature pigs (scale bar = 200 μm) **(B)**. Intestinal organoid monolayers of WZS pigs were cultured in OGM and ODM, respectively (scale bar = 100 μm) **(C)**. TEER of intestinal organoid monolayers was tested at 12, 24, 36, and 48 h after seeding **(D)**. TEER of intestinal organoid monolayers in Transwell inserts was calculated by subtracting the resistance of the blank group, and the final value in *Ω* cm^2^ = valid resistance value × membrane area (0.33 cm^2^). All experiments were in triplicate.

A physiological multicellular *in vitro* model of the intestinal epithelium is required for studying the function of the epithelial barrier, enteric pathogens’ infection, and mucosal immune responses in WZS pigs. To address this, adult WZS pigs (representative photographs of adult WZS pigs are shown in Figure 1A) were selected to establish intestinal organoid cultures. 3D intestinal organoids were differentiated from crypts isolated from the ileum of adult WZS pigs. Spheroidal 3D intestinal organoids formed after 4–7 days of culture in OGM (Figure 1B). To acquire adequate epithelial cells to generate a 2D monolayer, 3D organoids were passaged 2–4 times. The porcine 2D organoid monolayers can be generated both in commercial OGM and ODM (data are not shown); however, the differences between these two monolayers are unclear. To address this, 2D organoid monolayers derived from WZS pigs were generated in OGM and ODM, respectively. 3D organoids were dissociated into single cells by TrypLE™ Express. The cells were resuspended with OGM or ODM. The epithelial cells were seeded into a Matrigel-coated Transwell insert. Light microscope imaging suggested that, both in ODM and OGM, epithelial cells reach 100% confluency after 24 h (Figure 1C). The TEER of monolayers peaked at 36 h in culture, even if the monolayers in OGM showed a higher TEER than in ODM since 24 h in culture (Figure 1D). Our data indicate that 2D intestinal organoid monolayers of WZS pigs can be generated both in OGM and ODM. Monolayers in OGM and ODM exhibited a distinct growth pattern. OGM can support a faster and more complete confluence of 2D intestinal organoid monolayers.

### Epithelial cell types on 2D organoid monolayers in OGM

**Figure 2:**
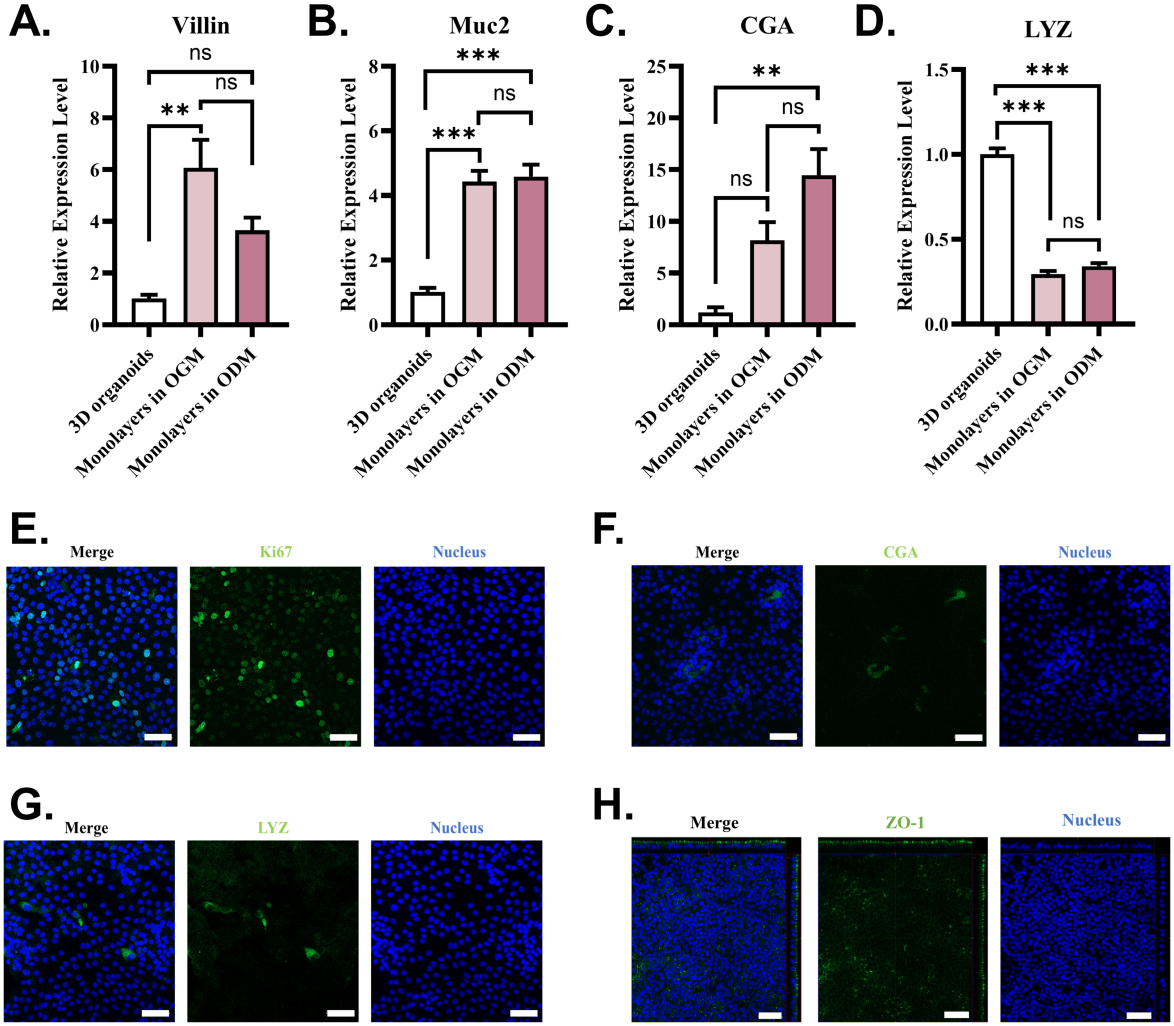
Epithelial cell types on 2D organoid monolayers in OGM The expression level of the marker gene of functional intestinal epithelial cells was evaluated using RT-qPCR **(A–D)**. Functional intestinal epithelial cell types in organoid monolayers of WZS pigs were identified through immunostaining specific to proliferating cells (Ki67+), enteroendocrine cells (CGA+), and Paneth cells (LYZ+) (scale bar = 50 μm) **(E–G)**. The formation of tight junctions in organoid monolayers of WZS pigs was tested through immunostaining to ZO-1 (scale bar = 50 μm) **(H)**. All experiments were in triplicate. *p*-values of <0.05 were considered statistically significant and indicated as **p < 0.01 and ***p < 0.001.

To interrogate the epithelial cell differentiation level in monolayers in two mediums, marker genes of enterocytes (Villin), goblet cells (Mucin2, Muc2), enteroendocrine cells (chromogranin A, CGA), and Paneth cells (lysozyme, LYZ) were detected using RT-qPCR. In contrast with 3D intestinal organoids, monolayers in both OGM and ODM showed a higher expressional level of Villin, Muc2, and CGA (Figures 2A–C). Interestingly, the mRNA level of LYZ decreased in monolayers (Figure 2D). Even if we observed differences in the mRNA level of Vilin and CGA in monolayers supported by two mediums, there were no significant differences between the two results. The data that suggested the formation of 2D organoids monolayer gave rise to changes in epithelial cell differentiation. OGM supports the faster and more complete confluence of 2D intestinal organoid monolayers. The RT-qPCR results indicate that 2D intestinal organoid monolayers in OGM also support the differentiation of epithelial cells. To identify epithelial cell types on 2D organoid monolayers of WZS pigs in OGM, functional epithelial cells, tight junctions, and proliferating cells were stained with IFA. The results indicate that proliferating cells are widely distributed in 2D organoid monolayers (Figure 2E). Enteroendocrine cells (CGA^+^) and Paneth cells (LYZ^+^) can also be observed in 2D organoid monolayers (Figures 2F,G). Through the Z-STACK imaging, ZO-1 can be identified on the apical side of 2D organoid monolayers (Figure 2H). These data demonstrated that the established 2D organoid monolayers of WZS pigs in OGM are multicellular models of intestinal epithelium with polarity.

### Organoid monolayers derived from WZS pigs are susceptible to TGEV infection

**Figure 3:**
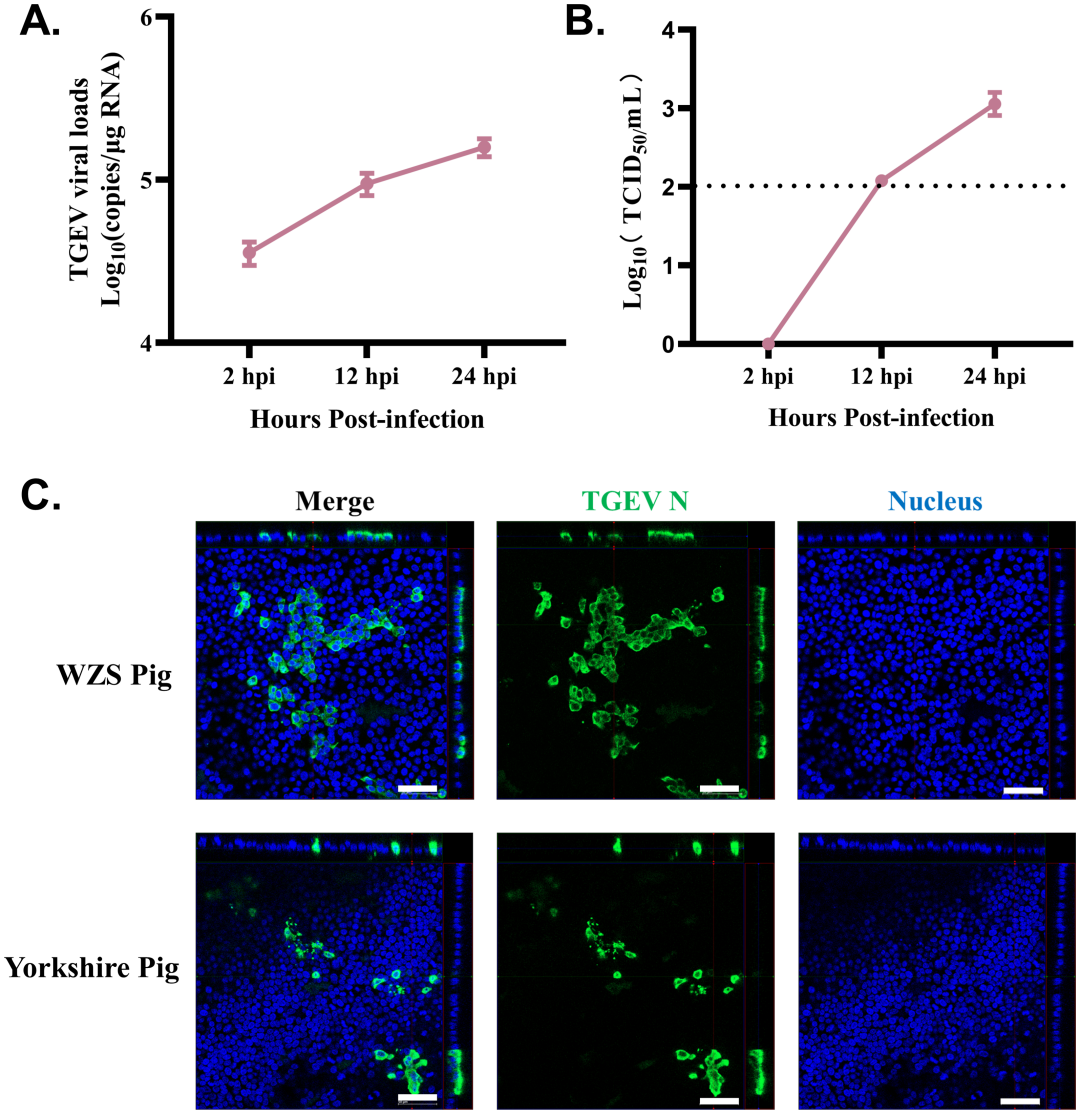
Organoid monolayers derived from WZS pigs are susceptible to TGEV infection Intestinal organoid monolayers of WZS pigs were infected with TGEV. TGEV viral load was evaluated through RT-qPCR at 2, 12, and 24 hpi **(A)**. Released TGEV was titrated via TCID50 assay at 2, 12, and 24 hpi **(B)**. The TGEV infection on organoid monolayers of WZS pigs and commercial Yorkshire pigs was confirmed through immunostaining specific to TGEV N (scale bar = 50 μm) **(C)**.

To assess the susceptibility of organoid monolayers of WZS pigs (in OGM) to TGEV, a widely studied enteric coronavirus infecting pigs, the TGEV Miller strain was used to infect organoid monolayers developed in this study. The TGEV viral gRNA loads in organoid monolayers increased from 2 to 24 hpi (Figure 3A). TGEV in supernatant was titrated using a TCID_50_ assay. Similar to gRNA load detection, TGEV viral titer was highest at 24 hpi for the timepoints sampled in this study (Figure 3B). In our previous study, we demonstrated that intestinal organoids (3D and monolayers) developed from commercial Yorkshire pigs are susceptible to TGEV (9, 10). Here, the immunostaining results showed that TGEV-infected cells can be identified in organoid monolayers derived from both WZS pigs and Yorkshire pigs (Figure 3C). These data indicated that organoid monolayers of WZS pigs are susceptible to TGEV and support the replication of TGEV.

### The gene transcriptional profiles of organoid monolayers upon TGEV infection

**Figure 4:**
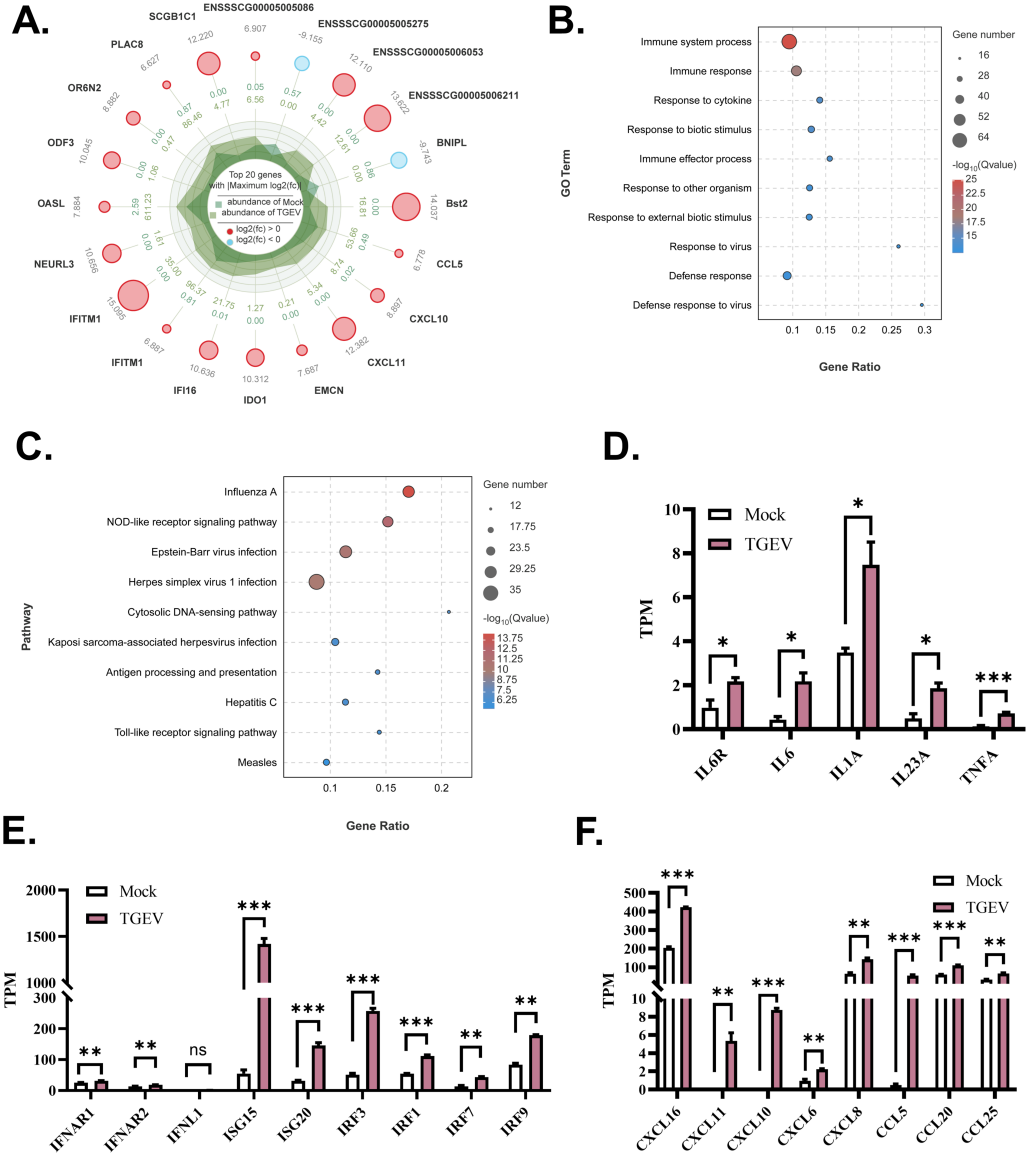
Gene transcriptional profiles of organoid monolayers upon TGEV infection The top 20 DEGs are shown in radar chart **(A)**. The DEG-enriched functions and pathways were evaluated via Gene Ontology Analysis and Kyoto Encyclopedia of Genes and Genomes Enrichment Analysis **(B,C)**. The expression level of inflammatory cytokines, interferon response-associated genes, and chemokines was analyzed to study TGEV-stimulated antiviral responses on intestinal organoid monolayers of WZS pigs **(D–F)**. The expression level of mRNA was normalized by transcript per million (TPM). All experiments were in triplicate. *p*-values of <0.05 were considered statistically significant and indicated as *p < 0.05 and **p < 0.01.

For investigating the responses of organoid monolayers of WZS pigs to TGEV infection, bulk RNA-seq was used to analyze the RNA landscape of TGEV-infected and control organoid monolayers in OGM. Upon TGEV infection, 369 upregulated genes and 39 downregulated genes were identified on organoid monolayers of WZS pigs. Among the top 20 DEGs, the expression of Bst2, CCL5, CXCL10, CXCL11, IFITM1, and OASL was upregulated in TGEV-infected organoid monolayers (Figure 4A). The expression of BNIPL, an apoptosis-associated gene, was inhibited in TGEV-infected organoid monolayers (Figure 4A). The Gene Ontology (GO) and Kyoto Encyclopedia of Genes and Genomes (KEGG) enrichment analysis indicated that TGEV infection-associated DEGs are mainly involved in the immune system process, immune response, response to cytokine, NOD-like receptor signaling pathway, and viral infection-related signaling (Figures 4B,C). The transcriptional levels of inflammatory cytokines (including receptors), interferon signaling-related genes, and chemokines were evaluated. The TGEV infection stimulated the transcription of IL6, IL6R, IL23A, and TNF-*α* (Figure 4D). TGEV significantly increased the mRNA levels of IFNL1, ISG15, ISG20, IRF3, IRF1, IRF7, and IRF9, indicating that TGEV stimulates IFNL1-associated interferon responses (Figure 4E). TGEV infection also promoted the expression of chemokines, including CXCL6, CXCL8, CXCL11, CXCL10, CXCL16, CCL5, CCL20, and CCL25 (Figure 4F). Overall, based on the TGEV infection model of organoid monolayers of WZS pigs, we demonstrated that TGEV infection stimulated innate immune responses at multiple levels and promoted the expression of chemokines on the intestinal epithelium, which mediate the chemotactic activities of immune cells in intestines upon TGEV infection.

## Discussion

The development of organoid techniques has brought about a breakthrough in biomedical research. The establishment of the intestinal organoids model of livestock also provided new insight into research surrounding veterinary medicine, animal development, and animal nutrition (11, 12). Porcine long-term primary multi-cellular intestinal organoids model has been well-established and used for studying pathogen invasion, intestinal epithelium development, pharmacology, toxicology, and nutriology (13–19). Here, we developed intestinal organoid models derived from the WZS pigs, a kind of unique miniature pig in the Hainan province of China. We also found that OGM supported a faster and more complete confluence of 2D intestinal organoid monolayers in WZS pigs. In a previous study, we established 3D and 2D porcine intestinal organoids derived from commercial pigs for studying viral infections (9, 10). OGM supported the growth and development of 3D intestinal organoids and the formation of 2D organoid monolayers. These studies indicated that OGM-supported intestinal organoid monolayers are an effective model of intestinal epithelial. However, we observed that 2D intestinal organoid monolayers are difficult to maintain in OGM for long-term study. According to the product information provided by the manufacturer, air–liquid interface (ALI) intestinal organoid monolayers can be cultured in ODM. Well-differentiated ALI intestinal organoid monolayer of livestock will be a more physiological model for veterinary medical research.

The epidemic of swine enteric virus threatens the pig industry and even human health. Because of non-intensive farming, up to now, the swine enteric virus infection on WZS pigs lacks research. Porcine intestinal epithelial cells are the main target cells of TGEV; therefore, intestinal organoids of WZS pigs provided a valuable model for studying virus–host interaction *ex vivo*. Consistent with the study on porcine intestinal organoids of commercial pigs, TGEV infection successfully activated type III interferon response and inflammatory pathways. Moreover, RNA-seq data indicated that TGEV infection stimulated the expression of important cytokines and chemokines on intestinal epithelial cells of WZS pigs. Interestingly, TGEV infection did not affect the expression level of tight junction-associated genes. This may be caused by the lack of immune cells in the monolayer model. Cytokines secreted by immune cells and the cytotoxic effect of immune cells upon viral infection may determine intestinal epithelium integrity (20). In TGEV-infected WZS pig intestinal organoid monolayers, the expression of SOX9, an intestine crypt transcription factor, was upregulated. This result suggested that TGEV infection facilitates the self-renewal of the intestinal epithelium, which is in accordance with the previous study (10). The WZS pigs were considered potential laboratory animals for studying human etiology, toxicology, and nutrition (1, 2, 21). The establishment of intestinal organoids derived from WZS pigs gives new insights into biomedical research based on the WZS pig model. The epidemiological data on coronavirus infection in WZS pigs still need to be improved. TGEV infection in intestinal organoids of WZS pigs also suggested the risk of spreading swine enteric coronavirus, which could pose a threat to laboratory animal management in future studies.

Overall, in this study, we established intestinal organoids derived from WZS pigs and evaluated their susceptibility to TGEV. As expected, TGEV infected intestinal organoid monolayers of WZS pigs and induced antiviral responses. These data will contribute to the utilization of WZS pigs as laboratory miniature pig models.

## Data Availability

The datasets presented in this study can be found in online repositories. The names of the repository/repositories and accession number(s) can be found: https://www.ncbi.nlm.nih.gov/, PRJNA1062093.

## Footnotes

^1^http://www.omicsmart.com
