# Supplementary material for: Wuzhishan miniature pig-derived intestinal 2D monolayer organoids to investigate the enteric coronavirus infection
Source: Front Vet Sci. 2024 Sep 25;11:1457719. doi: 10.3389/fvets.2024.1457719 (PMC11461462; doi:10.3389/fvets.2024.1457719)
Supplement: Supplementary file 1 [file Data_Sheet_1.PDF]

Supplementary Material

Supplementary Table1

Target gene	Primer/probe	Sequence (5' – 3')
Villin	Forward	TTGTAGCGGAGATGAGCGGGAGA
	Reverse	CGGGGAGTGATGACCAGGGTTTC
Muc2	Forward	GGCTGCTCATTGAGAGGAGT
	Reverse	ATGTTCCCGAACTCCAAGG
CGA	Forward	GACCTCGCTCTCCAAGGAGCCA
	Reverse	TGTGCGCCTGGGCGTTTCTT
LYZ	Forward	GGTCTATGATCGGTGCGAGT
	Reverse	AACTGCTTTGGGTGTCTTGC
