# Supplementary material for: Wuzhishan miniature pig-derived intestinal 2D monolayer organoids to investigate the enteric coronavirus infection
Source: Front Vet Sci. 2024 Sep 25;11:1457719. doi: 10.3389/fvets.2024.1457719 (PMC11461462; doi:10.3389/fvets.2024.1457719)
Supplement: Supplementary file 2 [file Data_Sheet_2.PDF]

Supplementary Material

Supplementary Figure1

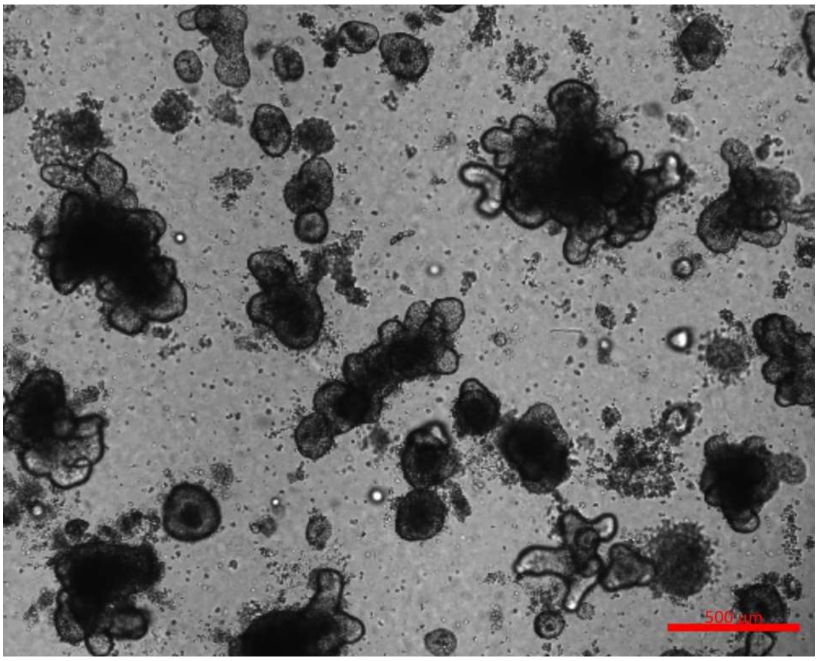

3D intestinal organoids derived from WZS pigs. Brightfield images of intestinal organoids of WZS pigs were captured after a 2-week culture (Scale bar = 500μm).
